# Fenugreek Seed Powder Nullified Aluminium Chloride Induced Memory Loss, Biochemical Changes, Aβ Burden and Apoptosis via Regulating Akt/GSK3β Signaling Pathway

**DOI:** 10.1371/journal.pone.0165955

**Published:** 2016-11-28

**Authors:** Asokan Prema, Arokiasamy Justin Thenmozhi, Thamilarasan Manivasagam, Musthafa Mohamed Essa, Mohammed D. Akbar, Mohammed Akbar

**Affiliations:** 1 Department of Biochemistry and Biotechnology, Faculty of Science, Annamalai University, Annamalai Nagar, Tamilnadu 608 002, India; 2 Department of Food Science and Nutrition, CAMS, Sultan Qaboos University, Muscat, Oman; 3 Ageing and Dementia Research Group, Sultan Qaboos University, Muscat, Oman; 4 Food and Brain Research Foundation, Chennai, Tamil Nadu 600094, India; 5 SMPT, NIAAA, National Institutes of Health, Rockville, MD, United States of America; USF Health Morsani College of Medicine, UNITED STATES

## Abstract

Alzheimer's disease (AD) is the most common form of dementia that mainly affects the cognitive functions of the aged populations. *Trigonella foenum-graecum* (L.) (fenugreek), a traditionally well utilized medicinal plant ubiquitously used as one of the main food additive worldwide, is known to have numerous beneficial health effects. Fenugreek seed extract could be able to inhibit the activity of acetylcholinesterase (AChE), a key enzyme involved in the pathogenesis of AD, and further shown to have anti-parkinsonic effect. The present study was aimed to explore the neuroprotective effect of fenugreek seed powder (FSP) against aluminium chloride (AlCl_3_) induced experimental AD model. Administration of germinated FSP (2.5, 5 and 10% mixed with ground standard rat feed) protected AlCl_3_ induced memory and learning impairments, Al overload, AChE hyperactivity, amyloid β (Aβ) burden and apoptosis via activating Akt/GSK3β pathway. Our present data could confirm the neuroprotective effect of fenugreek seeds. Further these results could lead a possible therapeutics for the management of neurodegenerative diseases including AD in future.

## Introduction

Dementia is an enervating disorder, which gradually declines the cognitive functions including memory, language, speech, orientation, judgment and learning capacity[1]. More than 36 million people are currently living with dementia worldwide and about 75% of this population that is, 27 million people, are estimated to be affected by Alzheimer's disease (AD) [2]. AD is accompanied by three main structural changes in the brain including (i) neuronal loss, formation and accumulation of hyperphosphorylated tau protein termed neurofibrillary tangles (NFT) and aggregation of β-amyloid (Aβ) peptides termed senile or amyloid plaques [3]. These changes are most prominent in the cholinergic system, particularly in hippocampus and cortex, which is closely associated with memory loss and cognitive dysfunction in AD[4].

Though the etiology of AD has not been discovered yet, both genetic and environmental factors play a vital role. A numerous lines of evidences implicated that aluminium (Al), an environmental toxin, acts as a causative factor for AD [2]. Al is the third most common element, comprising about 8% of the earth’s crust, exceeded only by oxygen and silicon. The ubiquitous presence of this element has so heavily contaminated the environment. Its exposure to humans is a massive possibility due to its presence in food, water, dust, air and medicines. Additional sources of aluminium are foods cooked and stored in aluminium utensils and foils [5] and usage of its compounds used in different processes including paper making, water treatment, fire retardant, fillers, food additives, colors and pharmaceuticals [6]. Al also enters into the human body by the consumption of corn, shellfish, yellow cheese, dairy products, spices, salt, breads, pastries, cakes, glace fruits, sausages, sugar-rich foods baking mixes, tea, herbs and cosmetics. Moreover Al compounds are used in antacids, phosphate binders, buffered aspirins, vaccines and allergen injections. People residing nearby areas of the cement factories are more prone to Al exposure as dispersed particulate matters contain high amount of aluminum [6]. Previous animal studies from our laboratory indicated that Al-induced neuropathological, neurochemical and neurobehavioral changes similar to AD [4,7,8]. Since Al is an environmental neurotoxin, whose exposure is get increased due to lifestyle changes and implicated in the pathogenesis of AD, the current experiment was designed.

Moreover excessive intake of Al causes the deposition of Aβ in the hippocampus and cortex, thereby causing learning and memory disorders in rats [7,8]. Aβ activates apoptotic cascades via accumulation of intracellular organelles such as endoplasmic reticulum (ER) or by binding directly to cell receptors, which may induce ER or mitochondrial stress [9]. The protein levels of amyloid precursor protein (APP) is increased in AD due to the dysregulated RNA processing with unspliced RNA species including myc box dependent-interacting protein 1, clusterin and presenilin-1 [10].Several signaling pathways such as MAPK [11], PI3K/AKT [12], NF-κB and Wnt pathways [13] might be involved in Aβ-induced neuronal apoptosis. Moreover Al could cause apoptosis by increasing the activation of caspase-3 and regulating the expressions of Akt and pGSK-3β [14].

*Trigonella foenum graecum* (L.) (fenugreek), a traditionally known medicinal plant widely distributed throughout the world including Asia (India and China), parts of Europe, North and South America, Africa and Australia [15]. Apart from the usage in frozen dairy products, spices, condiments, pickles, bakery products and beverages, fenugreek has also been reported to show antiviral, antimicrobial, antitumor, antioxidant, anti-inflammatory, antiapoptotic, hypotensive and antidepressant activities [16]. Traditionally, both the leaves and seeds are used as a medicine for the hypercholesterolemia and diabetes among Indian and Chinese population [17]. Satheeshkumar et al., [18] showed the inhibitory potential of fenugreek seed extract on the activities of acetylcholinesterase, a key enzyme involved in the pathogenesis of AD. Moreover oral treatment of fenugreek seed powder (FSP) (5%) reduced renal toxicity induced by aluminium chloride (AlCl_3_) in rats [19]. It also exhibited anti-parkinsonic effect by attenuating behavioral changes and inhibiting the activity of MAO-A and B [20] in rats. Glycosides based standardized fenugreek seed extract (SFSE-G) decreased apoptosis via modulation of Bax, Bcl-2 and caspase-3 against bleomycin induced experimental pulmonary fibrosis [21]. Few active compounds such as diosgenin, 4-hydroxyisoleucine and fibers of fenugreek attenuated insulin resistance in streptozotocin-treated rats and L6 myotubes via regulating AMPK- and AKT-dependent pathway in the liver [22].

In developing countries, Al exposure gets increased due to lifestyle changes, lack of public awareness and limited industrial waste management. Since 80% of the world population relies on the plant based medicine and fenugreek is extensively consumed as low cost condiment and generally accepted medicine, the neuroprotective effect of FSP was studied against AlCl_3_ induced toxicity by evaluating the cognitive impairments, levels of aluminium, activity of AChE and expressions of Alzheimeric (APP, amyloid β_1–40_&_1–42_, β and γ -secretases), pro-apoptotic (Bax, Bad, cyto c, caspases 3 and 9), anti-apoptotic (Bcl2 and Bcl–xL) and signaling (GSK-3β/Akt) markers.

## Materials and Methods

### Experimental procedures

#### Chemicals

Aluminium chloride, antirabbit-β-Amyloid, γ- and-β-secretase and amyloid precursor protein (APP) were purchased from Sigma-Aldrich, Bangalore, India and used in this study. Anti-rabbit-Bax, Bad, Bcl-2, Bcl–xL, cyto-c, pro and cleaved caspases-3,-9, GSK-3β, pGSK-3β (ser 9), pAkt (ser 473), Voltage-dependent anion channel (VDAC), anti- β actin (mouse) and horseradish peroxidase (HRP) conjugated goat anti-rabbit IgG were procured from Cell Signaling. All other chemicals used were of analytical grade.

#### Preparation of fenugreek seeds powder (FSP)

*Trigonella* seeds were purchased from the local market, Chidambaram, Tamilnadu, India, germinated and finely powdered. For the preparation of 2.5, 5 and 10% of FSP, about 2.5, 5 and 10 g of dried fenugreek seed powder were mixed with 97.5, 95.0 and 90.0 g of grounded rat food [19].A voucher specimen of the plant material (DBAU–1034) has been retained in the Department of Botany, Annamalai University, 608 002. We have done the qualitative analysis of chemical components and our products showed the presence of diosgenin, 4-hydroxyisoleucine, phenols and flavonoids.

#### Animals and treatment

Male Albino Wistar rats (200–225 g; 10–12 weeks age) were procured and maintained in Central Animal House, Rajah Muthiah Medical College & Hospital, Annamalai University under standard conditions [4]. The present study (Proposal No.1125) was approved by the local Animal Ethics Committee of the Institute (Reg. No. 160/1999/CPCSEA).

#### Phase I experiment

After 1 week acclimatization period, thirty six rats were randomized and divided into six groups of each containing six animals. Group I animals received saline and were considered as control. Group II rats were administered with AlCl_3_ (100mg/kg b.w., oral) for 6 weeks [23]. Though the dose of aluminum used to induce AD in rats was far higher than routine human exposure [2], it is correlated to human, who are exposed to extreme levels of aluminum under certain conditions e.g. occupational aluminium toxicity including welding, living near to the cement factories and dialysis encephalopathy [6, 24]. Based on the previous studies [23, 25], dose and duration, strain and age of the animals were selected to ensure the establishment of aluminium induced behavioral, biochemical, molecular and neuronal deficit, that resembles human AD. Group III rats were treated AlCl_3_ as group II and 2.5% FSP mixed in ground standard rat feed (i.e. 2.5 g of dry FSP in 97.5 g of ground rat food) [19] for 6 weeks. Group IV received AlCl_3_ as group II and 5% FSP mixed in grounded standard rat feed (i.e. 5 g of dry ground FSP in 95 g of ground rat food). Group V rats received AlCl_3_ as group II and 10% FSP mixed in grounded standard rat feed (i.e. 10 g of dry ground FSP in 90 g of ground rat food). Group VI animals received 10% FSP mixed in ground standard rat feed for 6 weeks. The dose of fenugreek seeds engaged in this experiment was chosen according to previous studies that has been subjected to nutritional and safety evaluation [19, 26]. Rao et al., [26] suggested that 5%, 10% and 20% administration of the FSP powder to the rats was equivalent to 1, 2 and 4 times, the therapeutic dose suggested for humans showed no toxicity. Food intake, water intake, and weight changes were recorded daily for 42 days. At the end of the experimental period, passive avoidance test was carried out. Then animals were anesthetized by using Ketamine hydrochloride (24 mg/kg body weight) (intramuscular injection), then the animals were sacrificed by cervical decapitation and the carcass were buried. After scarification, brain tissues (cortex and hippocampus) were excised and utilized for the determination of Al and AChE. No significant changes were found in the levels and activities of Al and AChE between 5 and 10% FSP co-treated rats. Hence, 5% of FSP has been used for further studies.

#### Phase II experiment

Forty eight randomly selected rats were divided into four (n = 12) groups: control, AlCl_3_, AlCl_3_+ FSP (5%) and FSP (5%) for 6 weeks. Neuroprotective effect of FSP against AlCl_3_induced experimental model of AD was determined by executing Morris water maze test and performing the protein expressions of Aβ biosynthesis related and apoptotic markers. In both the phases, animals were maintained in standard conditions (12/12 hour light/dark cycle, ~22°C temperature, and 60% humidity) with food and water *ad libitum* at home cage. Rats were acclimatized for 1 week prior to the start of the experiment. To evaluate the health status, measurement of body weight, observations of deviations from normal behavioral parameters and examination of physical appearance were performed daily basis. Moreover food and water intake were also measured daily. No abnormal signs and symptoms were observed throughout the experimental period. No abnormal signs and symptoms were observed throughout the experimental period. No toxic signs/mortality were observed throughout the experiment period.

#### Passive avoidance task

The apparatus consisted of two chambers (light and dark) with a metal grid floor. Both the chambers were separated by a wall that contains a door. The test was carried out on two consecutive days. In the acquisition trial, each and every rat was independently placed in the light chamber. Soon after entering into the dark chamber, an electric shock (40 V, 0.5 mA for 1 second) was delivered to the feet of the animal through the grid. The rat was immediately taken out from the apparatus and returned to the cage. Rat was placed again in the light chamber after 24 hours and the time taken to enter into the dark chamber was calculated as step-through latency. If the animal did not enter the dark chamber within a 5-minute test period, the test was ceased and the step-through latency was noted as 300 seconds [27].

#### Morris water maze

The apparatus consists of a large circular swimming pool (150 x 45 cm; water filled upto the depth of 30 cm at 28 ± 1°C), which is divided into four equal quadrants and a Perspex platform. During the acquisition phase, a small platform was placed about 1 cm above the water level. Each rat was subjected to four consecutive trials with a break of 5 min. Each and every animal was gently placed in the different quadrants for each trial, facing the wall of pool and permitted 120 s to locate the platform. Then, it was allowed to stay for 20 s in the platform. Animals were guided to reach the destination, if failed to reach the platform within 120 s and allowed to remain there for next 20 s. On day, 19 and 20, the animals were allowed to attend two consecutive training sessions. The mean time to reach the visual platform was measured as acquisition latency. On day 21 and 42, after AlCl_3_ administration, mean time to locate the hidden platform was recorded as first retention latency and second retention latency respectively [23].

#### Estimation of aluminum concentration

Both the hippocampal and cortical tissues were weighed and then added with poly-tetra-fluoroethene, 0.05 ml nitric acid and 0.2 ml H_2_O_2_ and incubated at 120°C for2 hours. The levels of aluminum were measured by atomic absorption spectrophotometer [7].

#### Assay of acetylcholinesterase activity

AChE activity assay kit was purchased from Bio Vision, INC, CA, USA and used to quantify the activity of AChE by ELISA method in hippocampus and cortex.

#### Expressions of proteins by western blot analysis

The hippocampus and pre-frontal cortex tissues were gently homogenized in 7 volume of cold suspension buffer and centrifuged (750 × g at 4°C; 10 min) to isolate the nuclear fraction and then at 10,000 × g at 4°C;20 min to separate the mitochondrial fraction. The pellets were re-suspended in cold buffer without sucrose and considered as the mitochondrial fraction. Supernatant was collected and centrifuged at 100,000 × g for 60 min at 4°C and the pellet obtained was then considered as cytosolic fraction [28]. Protein concentration in both the tissue fractions was analyzed by the method of Lowry et al., [29].

About 50 μg of total cellular protein were loaded on 10% of SDS-PAGE and transferred onto a Polyvinylidene fluoride membrane (Millipore) after separated. Membranes were incubated with the blocking buffer (with 5% non-fat dry milk powder) for 2 h to reduce non-specific binding sites and then incubated with APP, β-amyloid, γ- and β-secretases, Bax, Bad, Bcl-2, Bcl-xL, pro and cleaved caspase 3, caspase 9, cytoc, pAkt, pGSK-3β, tGSK-3β, VDAC (rabbit monoclonal; 1:250) and β-actin (rabbit monoclonal;1:1000) in TBST (5% bovine serum albumin in Tris-buffered saline and 0.05% Tween-20) and placed in a shaker at 4°C for overnight. Then membranes were hatched with secondary antibodies (IgG conjugated to horseradish peroxidase) at room temperature for 2 h. For 30 min, membranes were washed thrice with TBST. Final results were visualized by the chemiluminescence protocol(GenScript ECL kit, Piscataway, NJ, USA). Gel image analysis program was used for the densitometric analysis. The data were normalized using β-actin and anti-VDAC antibody as a cytosol and mitochondrial loading control respectively [30].

#### Data analysis

All data were expressed as mean ± Standard Error (SEM) of number of experiments. The statistical significance was calculated by one-way analysis of variance (ANOVA) using SPSS version 15.0 and the individual comparisons were obtained by Duncan’s Multiple Range Test (DMRT). A value of P< 0.05 was considered to indicate a significant difference between groups and the values sharing a common alphabet do not differ significantly with each other.

## Results

### FSP administration attenuates AlCl_3_ induced weight loss

**Fig 1:**
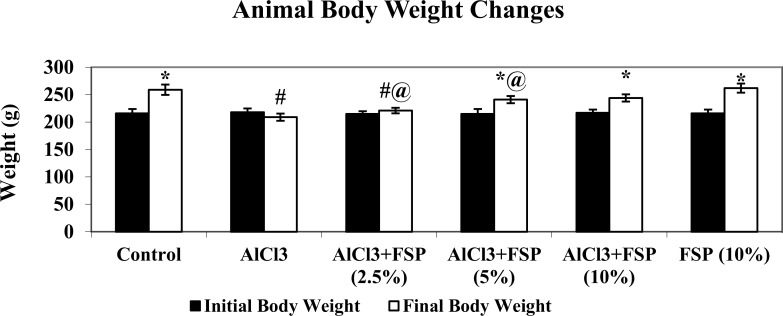
Rats induced with AlCl_3_ showed a significant (P<0.05) decrease in body weight when compared with control rats. Oral treatment with FSP to AlCl_3_ induced rats significantly (P<0.05) increased the body weight dose dependently. There are no significant changes in weight gain of FSP alone treated rats when compared with control rats. Data are expressed as mean ± SEM (one-way ANOVA followed by DMRT) for six rats in each group. Values not sharing the same symbols differ significantly.

Fig 1 shows the body weight changes in normal and experimental groups. Rats induced with AlCl_3_ showed a significant (P<0.05) decrease in body weight when compared with control rats. Oral treatment with FSP to AlCl_3_ induced rats significantly (P<0.05) increased the body weight dose dependently. There are no significant changes in weight gain of FSP alone treated rats when compared with control rats. The water intake (mL/d) and food intake (g/d) of rats in the control and experimental groups also showed no significant differences.

### FSP administration attenuates AlCl_3_ induced learning and memory impairments

Learning is manifested by organized behavioral changes, as a result of repetitive experience to the same stimulus environment and the conservation of learned behavior in over time is known as memory [31]. These processes cannot be measured directly, but could be observed by behavior changes under specialized conditions. Various behavioral test were employed to measure spatial memory (morris water maze, radial arm water maze and barnes maze), associative learning tasks (passive avoidance, fear conditioning), alternation tasks (Y-maze/T-maze), recognition memory tasks (novel object recognition), attentional tasks (choice serial reaction time), set-shifting tasks and reversal learning tasks. Measurement of behavioral changes is a more sensitive indicator of neurotoxicity during aluminium exposure [32].

**Fig 2:**
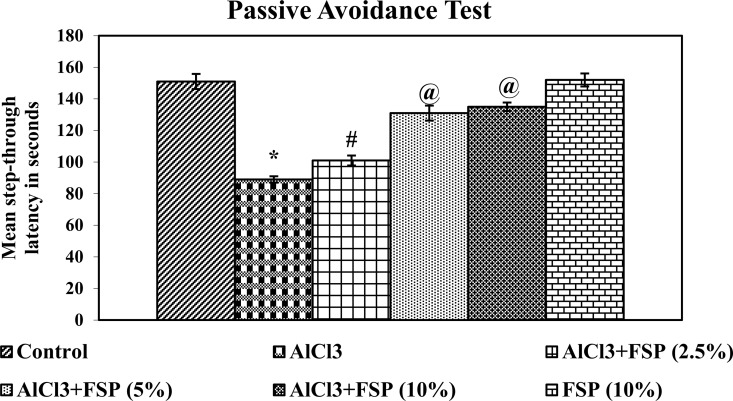
AlCl_3_ treated rats exhibited increased step-through latency (STL) in passive avoidance test. AlCl_3_ induced STL was reduced dose dependently by FSP co-treatment. Data are expressed as mean ± SEM (one-way ANOVA followed by DMRT) for six rats in each group. Values not sharing the same symbols differ significantly−*p < 0.05 compared to the control, ^#^p < 0.05 compared to the AlCl_3_ treated rats, ^@^p < 0.05 compared to the AlCl_3_ + FSP (2.5%).

In the passive avoidance task, the animal must learn to avoid or escape from aversive stimuli *i*.*e*., an electric shock exposure in darkness. All the nocturnal animals including rats naturally chooses only dark environment, but the animal has to suppress this tendency by remembering the negative stimulus. Rats treated with AlCl_3_ (Fig 2) showed a decreased step-through latency in passive avoidance task relative to control group, which indicates the memory impairment. On the other hand, co-treatment of FSP dose dependently and significantly reversed the AlCl_3_ induced memory and learning deficits as compared to AlCl_3_ alone treated rats. There was no significant difference found in the memory improvement between 5% and 10% FSP co-treated rats, but more significant than 2.5% FSP co-treated rats.

**Fig 3:**
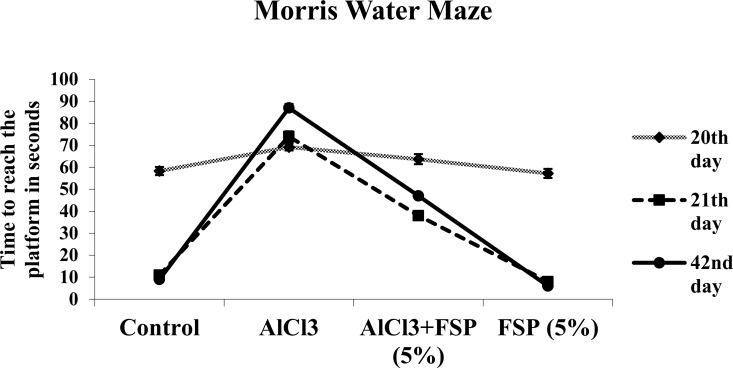
AlCl_3_ rats took more time to reach both the visible (on day 20) and hidden (on day 21 and 42) indicating memory deficits. Co-treatment of FSP (5%) significantly enhanced memory performance on day 20, 21 and 42 in both training and retention phase. Data are expressed as mean ± SEM (a repeated-measured ANOVA followed by DMRT) for six rats in each group. Values not sharing the same symbols differ significantly−*p < 0.05 compared to the control, ^#^p < 0.05 compared to the AlCl_3_ treated rats.

Morris water maze test is used to examine the age-related/ AD like conditions, because it is more specific for hippocampal function, one of the most affected regions in AD [33]. Rats are allowed to swim in a water tank filled with water and motivated to escape from the water by swimming to a hidden platform. The animals are learnt to locate the hidden platform by using spatial cues (posters or objects purposefully placed on the walls outside of the water maze). After several days, the time taken to locate the hidden platform by animals can be measured as spatial memory. Rats treated with AlCl_3_ took longer time to reach the visible platform than those of the control group on day 20, indicating memory deficits, whereas administration of FSP (5%) significantly enhanced memory performance on day 20 as compared to AlCl_3_-treatedgroup. Moreover AlCl_3_ treatment significantly diminished the 1^st^ and 2^nd^ retention latencies (on day 21 and 42 respectively) as compared to the control group. Chronic FSP (5%) treatment significantly enhanced both the retention latencies as compared to AlCl_3_ alone treated rats (Fig 3). A repeated measures ANOVA with a Greenhouse-Geisser correction determined that mean retension latency differed statistically significantly between time points (F (1.016, 371.806) = 38.101 p < 0.0005). Post hoc tests using the Bonferroni correction revealed that reduction in retension latency from training (20th day) to first retension latency was statistically significant (p < .005). However, second retension latency was not significantly different to first retension latency (p = 0.258).

### FSP amolerates AlCl_3_ induced Al overloading and AChE activity

**Fig 4:**
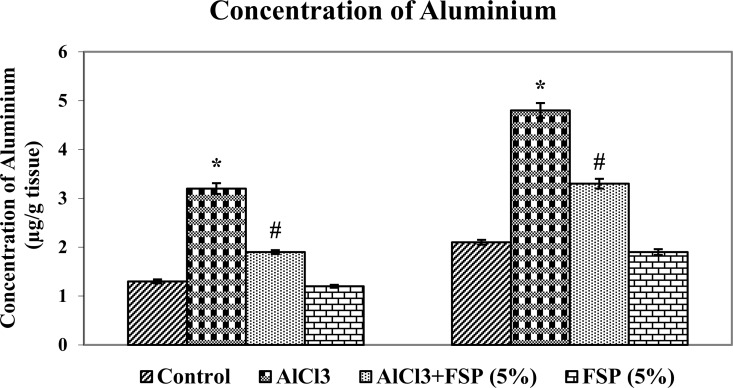
AlCl_3_ animals exhibited enhanced levels of Al in hippocampus and cortex. Cotreatment of FSP (2.5, 5 and 10%) dose dependently attenuated the AlCl_3_ mediated Al burden. Data are expressed as mean ± SEM (one-way ANOVA followed by DMRT) for six rats in each group. Values not sharing the same symbols differ significantly−*p < 0.05 compared to the control, ^#^p < 0.05 compared to the AlCl_3_ treated rats.

**Fig 5:**
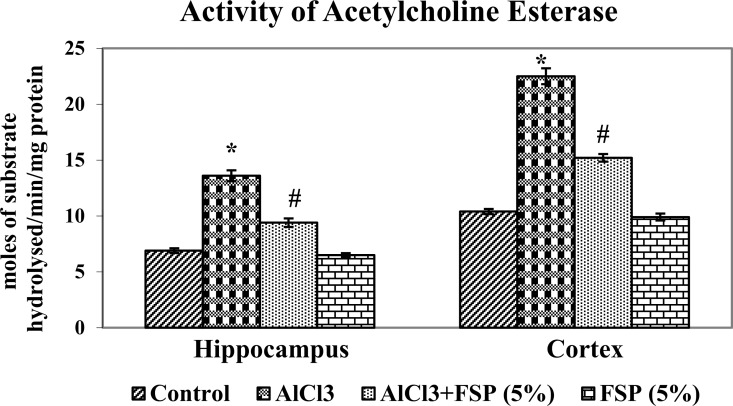
AlCl_3_ group showed significantly enhanced AChE activity in hippocampus and cortex. However, FSP (2.5,5 and 10%) co-treatment significantly attenuated the AChE hyperactivity in both regions of brain. It was observed that 5% and 10% FSP treatment showed similar reduction in Al levels and AChE activity, but more significant than 2.5% FSP. As a consequence, we have chosen the optimum dose (5% FSP) for our further study. Data are expressed as mean ± SEM (one-way ANOVA followed by DMRT) for six rats in each group. Values not sharing the same symbols differ significantly−*p < 0.05 compared to the control, ^#^p < 0.05 compared to the AlCl_3_ treated rats.

Researchers discussed the possible role of aluminium in AD for more than 50 years. Not only the AD patients and also the experimental animals overloaded with aluminum showed high concentrations of aluminium in the hippocampus and cortex [34]. Although aluminium is reported to accumulat in basal forebrain, brain stem and cerebellum, the cortex and hippocampus are the most vulnerable region for aluminium toxicity and essential for cognitive processes such as learning and memory. Cholinergic neurons and acetylcholine are linked to learning, memory, movement and blood flow control in the brain [35]. Aluminium induces memory impairment by decreasing cholinergic function, as measured by acetylcholinesterase (AChE) and choline acetyltransferase activities, the key enzymes involved in the degradation and synthesis of acetylcholine [36]. Aluminum chloride treatment significantly increased aluminum concentration (Fig 4) and AChE activity (Fig 5) in hippocampus and cortex as compared to control rats. However, FSP (2.5, 5 and 10%) co-treatment significantly attenuated the rise in aluminum overload and AChE hyperactivity in both regions of brain as compared to control rats. It was observed that 5% and 10% FSP treatment showed similar reduction in Al levels and AChE activity, but more significant than 2.5% FSP. As a consequence, we have chosen the optimum dose (5% FSP) for further studies.

### FSP nullifies AlCl_3_ induced Aβ biosynthesis and apoptosis

**Fig 6:**
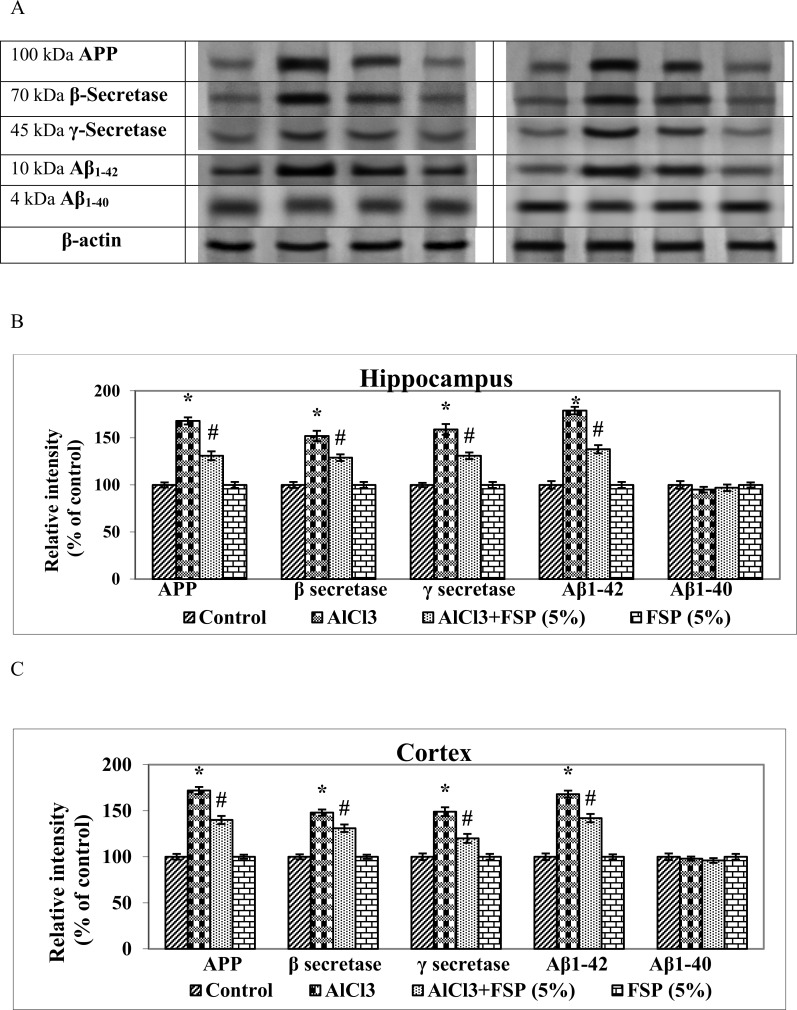
AlCl_3_ treatment significantly enhanced the protein expressions of APP, Aβ_1–42_, β and γ secretases and favours amyloid biosynthesis. Coadministration of FSP attenuated the AlCl_3_ mediated amyloid biosynthesis. Data are expressed as mean ± SEM (one-way ANOVA followed by DMRT) for three rats in each group. Values not sharing the same symbols differ significantly−*p < 0.05 compared to the control, ^#^p < 0.05 compared to the AlCl_3_ treated rats.

**Fig 7:**
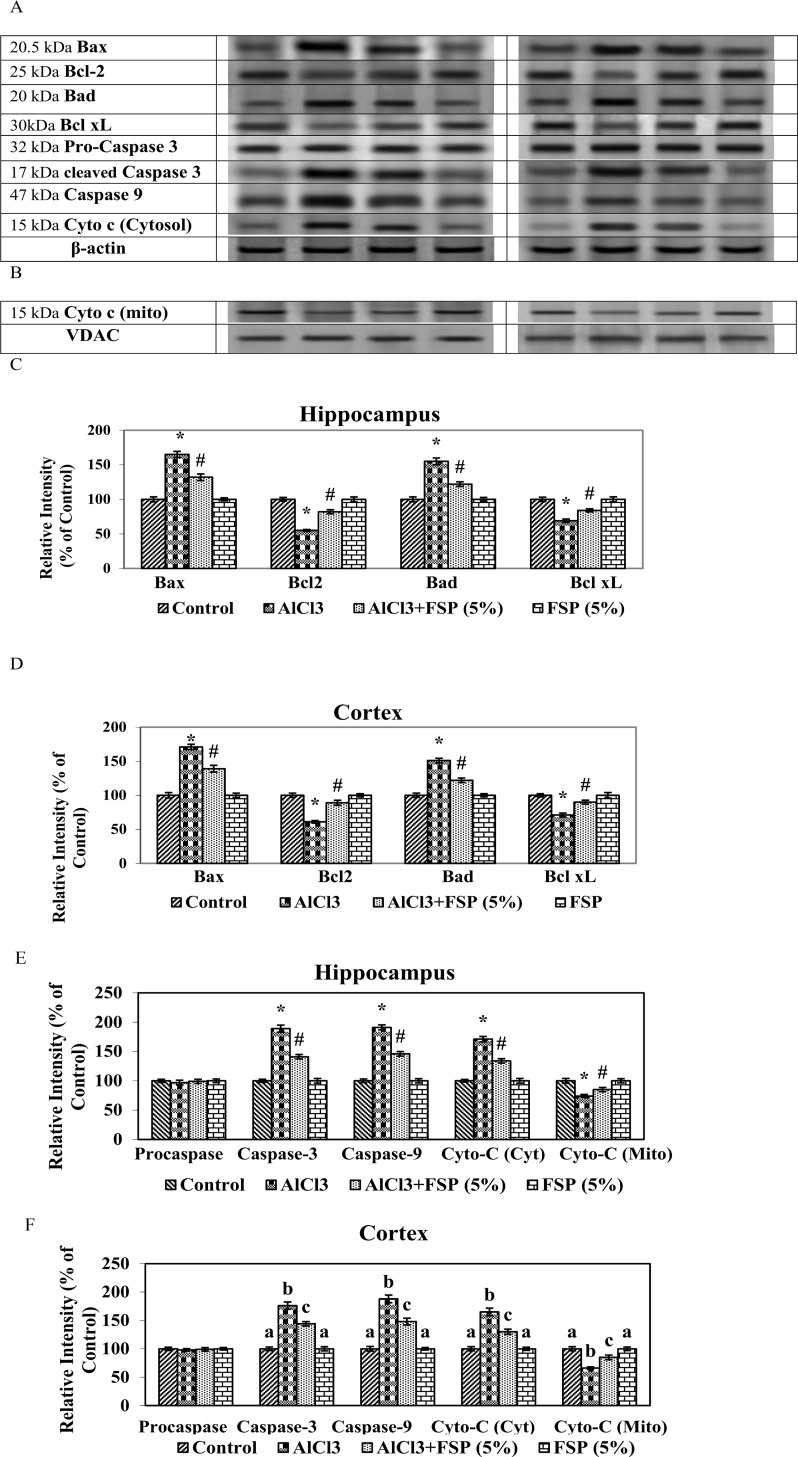
Chronic treatment of AlCl_3_ significantly increased the protein expressions of Bax, Bad, cyto c, caspases -9 and cyto c (mitochondrial fraction) and decreased the expressions of Bcl-2, Bcl—xL and cyto c (cytosolic fraction) in the hippocampus and cortex and favours apoptosis. However, FSP supplementation (5%) attenuated the AlCl_3_ induced apoptosis. No-significant changes in the expressions of pro-caspase-3 (32 kDa) were found in control and experimental groups. The activated caspase-3 (17 kDa) expression is enhanced following aluminum treatment and inhibited by the FSP co-treatment, which further proves the antiapoptotic property of FSP. Data are expressed as mean ± SEM (one-way ANOVA followed by DMRT) for three rats in each group. Values not sharing the same symbols differ significantly−*p < 0.05 compared to the control, ^#^p < 0.05 compared to the AlCl_3_ treated rats.

Results obtained from western blot analysis demonstrated the protein expression patterns of amyloid biosynthesis (Fig 6A, 6B & 6C) and apoptotic (Fig 7A, 7B, 7C, 7D, 7E & 7F) indices in hippocampus and cortex of control and experimental rats. Al is reported to enhance the Aβ burden in brain of experimental animals by directly influencing the Aβ biosynthesis or directly or indirectly affecting the Aβ catabolism [37]. Chronic administration of AlCl_3_ enhanced the protein expressions of APP, Aβ_1–42_, β and γ secretases as compared to the control group that would be in favor of Aβ plaque formation. Treatment of FSP (5%) to AlCl_3_ treated rats showed diminished expressions of APP, Aβ_1–42_, β and γ secretases.

Apoptosis is a prominent form of cell death in numerous neurodegenerative diseases like AD and Parkinson’s disease [38]. Recent studies from our lab showed that Al potentially induces apoptosis in brain by enhancing the expressions of Bax and caspases and by reducing the expressions of Bcl-2 [4, 27]. Chronic treatment of AlCl_3_ for a period of 6 weeks significantly increased (p < 0.005) the protein expressions of Bax, Bad, cyto c, caspases -9 and cyto c (mitochondrial fraction), whereas it decreased the expressions of Bcl-2, Bcl-xL and cyto c (cytosolic fraction) in the hippocampus and cortex. However, FSP supplementation (5%) attenuated the AlCl_3_ induced altered protein expressions. No significant changes were found in the hippocampal and cortical cytosolic expressions of pro-caspase-3 (32 kDa) of control, aluminum, aluminum/FSP and FSP-treated animals. Caspase-3 (17 kDa), one of the activated forms of caspase-3, expressed less incontrols and is present as an intense band in the aluminum-treated animals. Treatment with FSP significantly inhibits the cleavage of pro-caspase-3 to the active caspase-3.

### FSP reverses AlCl_3_ induced altered expressions of Akt/pGSK3β signaling markers

**Fig 8:**
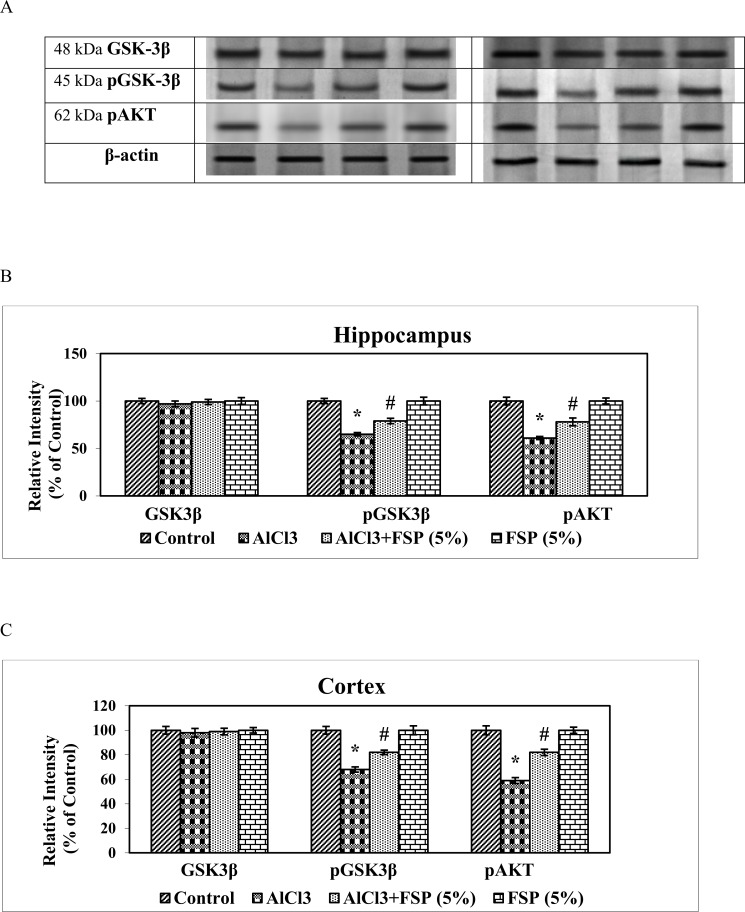
AlCl_3_ rats exhibited significantly lowered the expressions of pAkt and pGSK-3β in hippocampus and cortex. Western blot studies indicated that their expressions were significantly attenuated by co-treatment with FSP (5%). Data are expressed as mean ± SEM (one-way ANOVA followed by DMRT) for three rats in each group. Values not sharing the same symbols differ significantly−*p < 0.05 compared to the control, ^#^p < 0.05 compared to the AlCl_3_ treated rats.

Al may induce cell death by regulating a variety of signaling pathways. Its exposure induces dephosphorylation and deactivation Akt and activation of proapoptotic regulators such as Bad. Furthermore, it induces dephosphorylation and activation of GSK-3β, one of the key enzymes involving in control of apoptosis. Reduction of intrinsic Akt activity and activation of GSK-3β is associated with mitochondrial depolarization and permeabilization, cytochrome c release, and caspase-3 activation. By inhibiting Akt pathway, Al induces apoptosis. Rats treated with AlCl_3_ exhibited significantly lowered expressions of pAkt and pGSK-3β in hippocampus and cortex, whereas their expressions were significantly attenuated by co-treatment with FSP (5%). FSP alone treatment induced non-significant changes in the expression of pAkt, GSK-3β and pGSK-3β compared with the control group. These results indicate that FSP effectively reversed the AlCl_3_ induced neurotoxicity by augmenting the expressions of pAkt and pGSK-3β (Fig 8A, 8B & 8C).

## Discussion

Results of the present study showed that the intake of AlCl_3_ through drinking water significantly increased Al concentrations in hippocampus and cortex of rats that is in consistent with other reports [7,8]. In normal rats, the half-life of aluminium in brain is about 150 days and gets decreased to 55 days, upon receiving defroxamine, a metal chelator. Accumulated Al in brain can be mobilized by iron chelator such as deferoxamine chiefly via a carrier mediated mechanism to protect the brain from Al by effluxing across the BBB into blood [39, 40].Crude extracts of fenugreek derived with various solvents including methanol, ethanol, hexane, acetone, dichloromethane and ethyl acetate showed to have strong iron chelation activity [41] that are mainly due to the presence of various hydroxyl radicals in their constituents. Meghwal and Goswami [42] suggested that germinated fenugreek seeds exhibited more benefits than dried seeds, as germination increases the bioavailability of phenolic and flavonoid compounds in fenugreek. FSP treatment significantly reduced the Al bioavailability and accumulation in brain may be due to its strong metal chelating property.

Our results indicated that AlCl_3_ significantly reduced contextual memory in passive avoidance test and spatial memory in Morris water maze test. In the passive avoidance test, AlCl_3_ treated rats do not remember the aversive stimulus and enter earlier into the dark chamber associated with shock as compared to the control rats that could remember. In the Morris water test, Al over loaded rats showed less capacity to retrieve and retain the location of hidden platform with the help of spatial information even after several days. FSP co-administration reversed aluminium induced memory deficits, which indicate its memory enhancing effect. Saini et al., [43] reported that FSP reversed the memory deficits induced by scopolamine and diazepam.

Acetylcholinesterase (AChE) is the primary cholinesterase which hydrolyses the neurotransmitter acetylcholine into choline and acetic acid, a reaction that allows a cholinergic neuron to come back its resting state after activation. Each molecule of AChE degrades about 25000 molecules of acetylcholine (ACh) per second in both neural and non-neural tissues [44]. AChE activity is sensitive to exogenous factors including diets [45] and the presence of metal such as Al [7,8]. In the present study, chronic AlCl_3_ administration in rats showed significant increase in the brain AChE activity, which is in line with the previous studies [7,8, 46]. Al ions interact with the peripheral sites of AChE and modify its secondary structure and eventually enhanced its activity [47]. Co-administration of fenugreek seed extract to AlCl_3_ intoxicated rats showed the possible neuroprotection by reducing AChE activity. Inhibiting the activities of AChE increases the level of the ACh with positive effects on cognitive events [48]. Satheeshkumar et al., [18] demonstrated the *in vitro* AChE inhibitory activity of fenugreek and its active component, trigonelline. The pathophysiology of AD is multifaceted and involves amyloid-β (Aβ) deposition, tau pathology, oxidative stress, inflammation, mitochondrial and proteosome dysfunction, metal-Aß interactions that leads to profound loss of cholinergic neurons [49]. Drugs that potentiate central cholinergic function (such as donepezil, rivastigmine and galantamine) have confer only modest benefits during early stages of the disease. So additional non-cholinergic therapies such as anti-amyloid strategies, transition metal chelation, administration of growth factors, hormones, herbs, nonsteroidal anti-inflammatory drugs, antioxidants, lipid-lowering agents, anti-hypertensives, selective phosphodiesterase inhibitors, vitamins (E, B12, B6, folic acid) and agents that target neurotransmitter or neuropeptide alterations are urgently needed. Hence, it is possible that, memory enhancing activity of FSP might be partially through the inhibition of AChE.

According to “amyloid hypothesis”, the overproduction and accumulation of Aβ peptides represent an early and vital process in the pathophysiology of AD leading to the formation of neuritic amyloid plaques. Amyloid precursor protein (APP) is sequentially cleaved by a series of proteases including β- and γ-secretases. β-secretase chops the ectodomain of APP and produces an APP C-terminal portion, which is further spliced by γ-secretase within the trans-membrane domain resulting in the release of two C-terminal variants: Aβ_1–40_ or Aβ_1–42_ [50]. Aβ_1–40_ comprises approximately 90% of total secreted Aβ, but it aggregates much more slowly than Aβ_1–42_ [51]. Aβ in amyloid plaques consists mainly of the Aβ_1–42_ species, whereas vascular amyloid is composed primarily of Aβ_1–40_. In the present study, AlCl_3_ treatment favors amyloidogenesis by enhancing the expressions of pathological amyloid biosynthesis related markers including APP, Aβ_1–42_, β and γ-secretases. Wang et al., [52] reported that the administration aluminium maltolate of significantly augmented Aβ_1–42_ contents in cortex and hippocampus, although no changes were observed in the levels ofAβ_1–40_.Targeting Aβ production and assembly could be a vital therapeutic strategy for treating AD [53]. FSP significantly attenuated amyloidogenesis by modulating the expressions of APP, Aβ_1–42_, β and γ-secretases, which could inhibit Aβ production.

Al induces apoptosis in hippocampus and cortex mainly through the down-regulation of anti-apoptotic mediators and up-regulation of pro-apoptotic factors [54]. The Bcl -2 family of proteins controls the mitochondria-mediated intrinsic apoptotic pathway, which is classified into two groups: the anti-apoptotic proteins such as Bcl-2 and Bcl-xL and the pro-apoptotic proteins including Bax, Bad and Bak. The balance between pro- and anti-apoptotic Bcl-2 family proteins determines the survival or death of cells. Bcl-2 inhibits apoptosis by preventing the release of cytochrome c whereas, Bax induces apoptosis by dimerizing and inactivating the anti-apoptotic Bcl-2 proteins and enhancing cytochrome c release. Cytochrome c subsequently activates caspases, which finally leads to cell death. Caspases are the group of proteases that plays a vital role in the activation of apoptosis, necrosis and inflammation. Some caspases including caspase-9 acts as an upstream “initiator” in apoptosis by blending cell death stimuli to the downstream “effector” caspases such as caspase-3. In our study, chronic AlCl_3_ treatment significantly increased the expressions of pro-apoptotic markers such as Bax, Bad, caspases -3,-9 and reduced the expressions of anti-apoptotic indices such as Bcl-2 and Bcl-xL. Previous studies from our lab [4, 27] indicated that the AlCl_3_ administration enhanced the release of mitochondrial cytochrome c into the cytoplasm that triggers the activation of caspase-9, thereby activates the capsase-3 and finally results in apoptosis, which is consistent with present results. However, treatment with FSP prevents AlCl_3_ induced apoptosis by reducing the expression of Bax, active caspases -3,-9, cytosolic cyto c and increasing the expression of Bcl-2 and preventing the release of mitochondrial cyto c, which is concordant with previous reports [21,55].

Kinase signaling pathways play a critical role in the regulation of cellular processes including apoptosis. Apoptosis in neurons are regulated by three important kinase pathways: c-Jun N-terminal kinase (JNK) pathway, protein kinase B (Akt) and glycogen synthase kinase-3 (GSK3) pathway [56,57,58]. The activation of Akt pathway promotes cell survival in many neuronal cell types [59, 60], while its inhibition promotes neuronal cell death [61]. Gelsolin is preteolytically cleaved in AD brains, which mediated activation of PI3K/Akt pathway is crucial [62].

In the present study, AlCl_3_ treatment down-regulated the expression of pAkt, whereas co treatment of FSP up-regulated pAkt, which is the active form of Akt. Activated Akt is believed to suppress apoptosis through regulation of the Bcl-2 family members including Bad [63], caspase-9 [64] and GSK-3β [65]. Activation of Akt leads to the down-regulation of GSK-3β by inducing the phosphorylation at Ser9 [66]. GSK-3β, a serine/threonine protein kinase, is one of the major tau kinase that involves in the phosphorylation of Tau protein, which in turn forms neurofibrillary tangles and amyloid plaques during AD [67]. The activity of GSK-3β is regulated by phosphorylation at Ser 9 and Tyr 216 [68, 69]. Ser 9 phosphorylation inhibits its kinases activity, whereas Tyr 216 phosphorylation is required for its full activity. In this study, we found that aluminium exposure decreased the expressions of Ser 9 pGSK3β, thereby enhancing the kinase activity of GSK3β and tau hyperphosphorylation, which is consistent with previous finding [70]. 5% FSP cotreatment enhanced the expressions of Ser9pGSK3β, thereby suppressing the kinase activity of GSK3β and tau hyperphosphorylation. Natural products are said to be beneficial for neuroprotection [71]. Our findings support the possibility of fenugreek to be used in AD treatment.

## Conclusion

As to conclude, our results demonstrated that FSP suppresses the AlCl_3_ induced memory and learning impairments, Al overload, AChE hyperactivity, Aβ burden and apoptosis via activating Akt/GSK3β pathway, which may be due to the synergistic action of its active components. However extensive research is needed to confirm the anti-alzheimeric effect of individual active components of fenugreek against various models of AD, before entering into the clinical trials.

## Supporting Information

S1 Dataset The data set and supporting information for Figs 1, 2 and 3. (XLSX)

S2 Dataset The data set and supporting information for Figs 4 and 5. (XLSX)

S3 Dataset The data set and supporting information for Fig 6. (XLSX)

S4 Dataset The data set and supporting information for Fig 7. (XLSX)

S5 Dataset The data set and supporting information for Fig 8. (XLSX)
